# A biomechanical analysis of strength and motion following total shoulder arthroplasty

**DOI:** 10.4103/0973-6042.39579

**Published:** 2008

**Authors:** John W. Sperling, Kenton R. Kaufman, Cathy D. Schleck, Robert H. Cofield

**Affiliations:** Department of Orthopedic Surgery, Mayo Clinic, Rochester, MN, USA

**Keywords:** Biomechanics, motion, strength, total shoulder arthroplasty

## Abstract

**Background:**

The primary goal of total shoulder arthroplasty (TSA) has traditionally been pain relief and motion improvement. The literature contains multiple studies that have documented the restoration of motion and consistent pain relief following the procedure. However, there has been little attention placed on strength following TSA. Therefore, the purpose of this study was to determine in an objective manner whether strength and motion improve with shoulder arthroplasty and over what time course this may occur.

**Materials and Methods:**

Between April 2002 and January 2004, 15 patients who underwent TSA for osteoarthritis had biomechanical strength testing preoperatively, at 6 and 12 months postoperatively. The mean age at the time of TSA was 66 years (range, 52-82). None of the patients had full thickness rotator cuff tears at the time of surgery. Peak forces for shoulder flexion, extension, abduction, internal rotation and external rotation strength were recorded. In addition, patients had shoulder range of motion measurements performed.

**Findings:**

Range of motion improved significantly with TSA from preoperative to 6 months postoperative: flexion 104° to 147° (*P* = 0.0034), abduction 86° to 145° (*P* = 0.0001), internal rotation 43° to 54° (*P* = 0.0475) and external rotation 25° to 50° (*P* = 0.0008). There was minimal improvement in range of motion from 6 to 12 months. In contrast, there continued to be improvements in strength from the 6 month to the 12 month postoperative time frame: extension 18.3 kg to 22.4 kg (*P* = 0.006), abduction 11.3 kg to 12.8 kg (0.0474) and external rotation 8.8 kg to 10.1 kg (*P* = 0.016). Despite these improvements, compared to normative values, there continued to be relative weakness of the shoulder following TSA.

**Interpretation:**

The data from this study suggest that recovery of strength and motion follow different time frames after TSA. The results of this study may allow the surgeon to more accurately discuss with the patient over what time course strength and motion may return. In addition, this study raises important questions in regard to the current rehabilitation program used after shoulder arthroplasty and whether development of new protocols may improve the functional outcome from surgery.

**Level of Evidence:**

Level 2

The primary goal of total shoulder arthroplasty (TSA) has traditionally been pain relief and motion improvement. There has been, however, little attention placed on strength after TSA. The few studies that report objective strength testing are limited in regard to both the directions of strength evaluation as well as testing at variable time periods.[1–3] There is minimal data on whether an improvement in strength and motion occurs after TSA. Therefore, the purpose of this study was to determine in an objective manner whether strength and motion improve with shoulder arthroplasty and over what time course these changes may occur.

## MATERIALS AND METHODS

Between April 2002 and January 2004, 15 patients who underwent TSA for osteoarthritis by the senior author underwent biomechanical strength testing preoperatively, at six months postoperatively and 12 months postoperatively. The mean age at the time of TSA was 66 years (range, 52-82). There were nine men and six women. All procedures were done through a deltopectoral approach. The subscapularis was incised through the tendon in seven patients and repaired with suture. In eight patients with significant restriction of external rotation, the subscapularis was incised off bone and repaired with suture in a medial position. None of the patients had full thickness rotator cuff tears at the time of surgery. The day following the surgery, active motion was started for the hand, forearm and elbow. In addition, a passive range of motion (ROM) program was started for the shoulder. Active assisted motion was commenced at six weeks, with isometrics starting at eight weeks and elastic strap strengthening at 12 weeks.

All patients underwent strength testing as outlined by Andrews *et al*.[4] Peak forces for shoulder flexion, extension, abduction, internal rotation and external rotation strength were recorded. A Chatillon hand held MSE 100 Dynamometer (AMETEK, Inc.) was used to collect isometric strength data of the shoulder. The subjects were tested in the supine position using standardized test positions as described by Andrews.[4] The strength was measured in kilograms. In addition, normative values for age and gender obtained by Andrews[4] were used to calculate the percent of normal strength. Submaximal practice trials were performed to familiarize and orient the subject to the action being requested by the evaluator. The subject was then asked to provide maximal effort for approximately 4 sec. Three trials were be performed. Patients also had shoulder ROM measurements performed by the same examiner with the user of a goniometer. Testing was performed by one of two specially trained physical therapists working in the Motion Analysis Laboratory. Tester reliability was obtained on four normal individuals prior to beginning the study.

### Statistics

A paired t-test was used to assess baseline to six month, six month to 12 month and baseline to 12 month changes in strength and motion. The strength measurements assessed were flexion, extension, abduction, internal and external rotation. The motion measurements assessed were flexion, abduction, internal and external rotation. Analyses were performed with the use of SAS version 9.13 (SAS Institute Inc, Cary, North Carolina, USA). The level of significance was set at *P* < 0.05.

## RESULTS

### Strength

**Figure 1:**
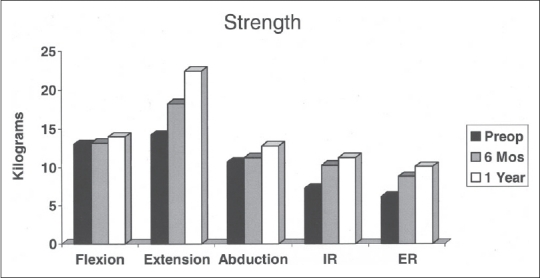
Graph demonstrating changes in strength at the three time points: preoperative, 6 months postoperative and 1 year postoperative

There were improvements in external rotation and internal rotation only comparing preoperative strength to that assessed at six months [Table 1, Figure 1]. There was significant improvement in extension, abduction and external rotation strength during the interval from six to 12 months. At the 12 month mark, there were significant improvements in abduction, internal rotation and external rotation compared to preoperative values. Despite improvement, one can see that compared to normative values, the shoulders continued to have decreased strength.

**Table 1:** Strength

	Strength kg (% normal)			*P*-value		
	Preoperative	6 months	1 year	Pre-op to 6 months	Pre-op to 12 months	6-12 months
Flexion	13 (53)	13.2 (61)	14 (62)	0.8672	0.4768	0.3734
Extension	14.2 (50)	18.3 (69)	22.4 (83)	0.092	0.0036	0.0006
Abduction	10.7 (51)	11.3 (58)	12.8 (64)	0.7095	0.3629	0.0474
Internal rotation	7.3 (43)	10.3 (66)	11.3 (71)	0.0456	0.0225	0.0577
External rotation	6.2 (44)	8.8 (67)	10.1 (73)	0.0061	0.0012	0.016

### Range of motion

**Figure 2:**
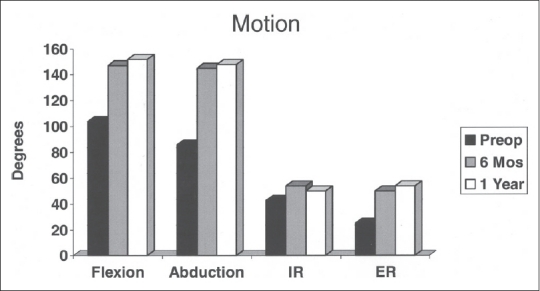
Graph demonstrating changes in motion at the three time points: preoperative, 6 months postoperative and 1 year postoperative

The data from the study indicate that there was significant improvement in motion in all directions tested from preoperative to 6 months with TSA [Table 2, Figure 2]. The data also show that there was minimal improvement in motion from 6 to 12 months. The only parameter that increased significantly was external rotation by 4° in this time interval. At the 12 month mark, there was significant improvement in all directions compared to preoperative values except internal rotation.

**Table 2:** Motion

	Degrees			*P*-value		
	Pre-op	6 months	1 year	Pre-op to 6 months	Pre-op to 12 months	6-12 months
Flexion	104	147	152	0.0034	0.0001	0.0906
Abduction	86	145	148	0.0001	0.0001	0.1209
Internal rotation	43	54	50	0.0475	0.116	0.835
External rotation	25	50	54	0.0008	0.0001	0.0348

## DISCUSSION

The data from this study suggest that recovery of strength and motion follow different time frames after TSA. There was minimal improvement in ROM after the first six months postoperatively. However, there continued to be significant improvements in strength from the sixth month to the 12 month postoperative time frame. In addition, compared to normative values, there continued to be relative weakness of the shoulder.

The data from this study may allow the surgeon to more accurately tell the patient over what time course strength and motion may return. In addition, this information may better guide surgeons in regard to the limitations that they set for their patients following shoulder arthroplasty. Lastly, the data from this study raises an important issue in regard to the current rehabilitation program used after shoulder arthroplasty and whether development of new protocols may improve the functional outcome from surgery.
